# Measured and perceived effects of audit and feedback on nursing performance: a mixed methods systematic review protocol

**DOI:** 10.1186/s13643-019-0956-1

**Published:** 2019-02-01

**Authors:** Émilie Dufour, Arnaud Duhoux, Jolianne Bolduc

**Affiliations:** 1 Faculty of Nursing, Université de Montréal, C.P. 6128 succ. Centre-Ville Montréal Marguerite-d’Youville Campus, Montréal, QC H3C 3J7 Canada; 2 CR-CSIS (Centre de recherche Charles-Le Moyne - Saguenay-Lac-Saint-Jean sur les innovations en santé) Université de Sherbrooke, Longueuil Campus, Longueuil, QC J4K 0A8 Canada; 3 ESPUM (École de santé publique de l’Université de Montréal), Université de Montréal, Montréal, QC H3N 1X9 Canada

**Keywords:** Nursing audit, Systematic review, Quality improvement

## Abstract

**Background:**

The use of audit and feedback (A&F) interventions in health care has been demonstrated to generally be effective on medical teams. However, literature suggests that the response of nurses to this type of intervention may differ from that of other types of health professionals, in relation to their roles, power, and to the configuration of nursing care activities. To our knowledge, no review has been conducted on A&F interventions with nurses. The objective of this systematic review is to examine the evidence of measured and perceived effects of A&F interventions on nurses’ performance.

**Methods:**

A mixed methods systematic review design with thematic and narrative synthesis is used. Studies reporting quantitative and qualitative data on the effects of A&F interventions specific to nursing care are considered for inclusion. Studies will be appraised for quality using the Mixed Methods Appraisal Tool. Quantitative and qualitative data will be summarized in narrative and tabular form and will be synthetized using a segregated methodologies approach.

**Discussion:**

Results will describe the characteristics of A&F with nurses, as well as the measured and perceived effects specific to nursing care. The associations between the characteristics and the effects as well as the concordance between measured and perceived effects will be presented. We anticipate that combining the evidence from qualitative and quantitative studies will allow us to provide relevant insight that can inform the design of better suited A&F interventions for nurses. Audit and feedback interventions demonstrate potential for improving the performance of nursing care. As their effectiveness varies greatly depending on the context and the professionals involved, a better understanding of the associations between its characteristics and the measured and perceived effects is valuable for improving the effectiveness of A&F.

**Systematic review registration:**

PROSPERO CRD42018104973

**Electronic supplementary material:**

The online version of this article (10.1186/s13643-019-0956-1) contains supplementary material, which is available to authorized users.

## Background

Scientific literature on quality improvement in healthcare highlights the effectiveness of audit and feedback (A&F) [1–5]. A&F is an intervention comprising of two components: (1) evaluation of the performance (audit) and (2) feedback to the professionals. Most of the literature pertaining to A&F involves physicians [1, 5–7]. However, some authors suggest that the response of nurses to this type of intervention may differ from that of other types of health professionals [8] in relation to their roles, power, and the configuration of nursing care activities [9–11]. Since nurses often work in teams, quality improvement involves changes both at individual and collective levels. Collective improvements, requiring communication, cohesion, and coordination within the nursing team [11, 12], make interventions with this group of professionals particularly challenging.

A&F should focus on elements of practice over which professionals perceive to have control and to be accountable for [1, 4], which has been suggested to be a current limitation in A&F interventions involving nurses [9–11]. Although many studies report the development and use of nursing-sensitive indicators, the access to measurable process indicators is currently lacking [13–16]. The use of process indicators, as they are more actionable and proximal to nursing care activities, could result in higher improvement rates of nursing performance [16]. The nature of the indicators reported to nurses could therefore influence the overall effectiveness of A&F. In order to design A&F interventions that are suitable for nursing activities, it is relevant to gather the current state of evidence on A&F targeting nurses.

To our knowledge, no systematic review has been conducted on A&F interventions with nurses. Mixed methods systematic reviews are a method of synthesizing knowledge which aims to combine several types of data concerning the same subject in order to present an integrated synthesis of the results produced through different methodologies [17]. A better understanding of how A&F unfolds in nursing practice settings could allow us to implement design elements according to the care settings and professionals involved. Given the paucity of literature on the effectiveness of A&F interventions with nurses [5] and the difficulty of quantitatively measuring nursing-specific effects [14, 18], it is relevant to study both the measured and perceived effects of this type of intervention. Performance, in the current paper, is addressed in a comprehensive way [19] and refers to dimensions of quality, effectiveness, and security.

### Characteristics of A&F interventions

Although the efficacy of A&F interventions has been well demonstrated, it is well acknowledged that it has large variations depending on certain characteristics [1, 20, 21]. Some authors have identified A&F characteristics which are more likely to modify its delivery and would therefore explain those variations [1, 2]. These characteristics relate to the form, content, and frequency of interventions [2]. Until now, no consensus has been established to provide best practices for developing A&F interventions. The effectiveness of A&F may also be influenced by, among other things, the type of professional, the context of the intervention, and levels of baseline performance [5].

### Measured effects

The effects of A&F interventions are usually assessed using quantitative benchmarks in the form of indicators, which monitor the action around the dimensions of performance [5]. As such, the structure-process-outcome triad developed by Donabedian [22] is a quality of care assessment framework designed to include all dimensions of performance of a health organization. To Donabedian [22], the structure component refers to all the elements relating to the framework in which care is provided, including both elements related to environment and those related to staff characteristics. The process component encompasses all the acts performed by the professional in their care delivery activities as well as the way these activities are performed. The outcome component refers not only to the effects of care on the health status of populations [22], but also to organizational outcomes, including the volume of care provided by a health facility [23]. In this review, this triad will be used to extract and analyze data from quantitative studies.

### Perceived effects

Quantitative evaluation of these three categories of indicators requires access to data [24]. In nursing, access to data that are both valid and reliable that allows nursing indicators’ measurement is an issue [18, 25–27]. At present, the measurable effects of A&F interventions on nursing performance have some limitations, such as variable access to reliable nursing-specific indicators [14–16]. Hence, it seems highly relevant to review the perceived effects associated with this type of intervention, which are generally evaluated through qualitative studies. Given the small amount of literature related to nurse-specific effects of AF [5], the inclusion of perceived effects is intended to provide a better understanding of how this type of intervention unfolds with nurses influences the perception of their performance. Murray et al. [28] proposed a framework for describing and evaluating the effects of implementing a complex intervention, such as A&F, within a health organization from the perspective of the professionals involved. We believe this framework provides a global and relevant perspective to better understand the actions of the professionals involved in the implementation of a complex intervention. According to this approach, the effects perceived by professionals when implementing an intervention in their practice relate to four areas. The first area, that of coherence, relates to the meaning and value that participants place on intervention [28]. The second area, that of cognitive participation, refers to the commitment and motivation that the implementation of the intervention entails for participants. The third area, that of collective action, refers to the actions that intervention requires in participants’ practice changes, for example, in terms of time and training. The last area, the reflexive domain, refers to the perceived effects of changes in practice, for example, in improving the care provided [28]. The assessment of these domains makes it possible to consider the context of implementation of the intervention and its effects on the response of the participants [28]. This framework will be used in this review to ensure the systematic nature of the data extraction and analysis from qualitative studies.

### Objective and research questions

The main objective of this review is to identify and appraise the existing evidence on the effects of A&F on nursing performance. It aims to answer the following questions:

1. What are the main features of A&F with nurses?
2. What are the measured effects of A&F on nurses’ performance?
3. What are the perceived effects of A&F on nurses’ performance?
4. What are the associations between the features of A&F and the measured and perceived effects?
5. What is the concordance between the measured and perceived effects of A&F with nurses?

## Methods

Considering mixed methods reviews’ approaches are still emerging, we will conduct the data extraction, analysis, and synthesis using a various range of guidelines [17, 29–33]. The specific contributions and applications of these guidelines are presented in each section of the method. The Preferred Reporting Items for Systematic Reviews and Meta-Analyzes (PRISMA) grid [34] will be used as a reference for the preparation and writing of this review. This mixed methods systematic review is registered with the International Prospective Register of Systematic Reviews (PROSPERO) CRD42018104973.

### Outcomes

The primary outcome will include any measured or perceived effect of the AF intervention on nursing-related dimensions of performance, such as quality and security of care and effectiveness. A measured effect is defined as any variable that is reported objectively and assessed through quantitative methods. A perceived effect is defined as any variable that is subjectively reported and assessed through qualitative methods.

### Eligibility criteria

Criteria have been developed to specifically address the research questions. Thus, studies that meet the following criteria will been included: (1) A&F intervention corresponding to a feedback of the performance to nurses over a given period of time [1], (2) focusing on an A&F intervention with nurses or health professionals including nurses and with specific effects on nurses, (3) describing characteristics of the A&F intervention, and (4) presenting an A&F intervention related to at least one effect related to a dimension of performance. The literature that will meet the following criteria will be excluded: (1) providing feedback to nursing students and (2) focusing on an A&F intervention targeting patients.

### Information sources

The strategy will be carried out in three stages. First, an initial search will be performed from CINAHL and MEDLINE to test the identified keywords and descriptors [17]. Then, a second search will be carried out based on the keywords and descriptors identified in all the selected databases. Then, the list of references of identified studies will be analyzed to identify additional articles. Authors recognized as experts in the field will be contacted by email to obtain any additional relevant study related to the research topic [35]. Online periodicals relevant to the area of interest will be identified and searched for additional and non-indexed articles in the databases [35]. Publications in English and French will be considered with no time restriction. The following databases will be used: CINAHL, Cochrane Controlled Register of Trials, Embase, MEDLINE, PubMed, Scopus, Web of Science, PsycINFO, and Global Health. The ProQuest database will be considered for tracking unpublished studies. Quantitative studies will include randomized controlled trials, nonrandomized studies, and descriptive studies [36]. Qualitative studies will include phenomenological studies, grounded theorization, ethnography, action research, case studies, and descriptive qualitative research [36]. Mixed methods studies, if any, will be considered.

### Search strategy

The following concepts will be sought: “feed back” OR “action planning” OR feedback OR audit OR audits AND nurs* AND performance OR quality OR effectiveness OR security OR efficacy. The following descriptors will be used: (MH “Feedback”) OR (MH “Audit”) OR (MH “Nursing Audit”) OR (MH “Clinical Audit”) AND (MH “Nursing Role”) OR (MH “Nurses +”) OR (MH “Nursing Care +”) AND (MH “Quality of Health Care”) OR (MH “Quality Improvement”) OR (MH “Clinical Effectiveness”) (see Additional file 1). Search equivalents terms in French will be sought.

### Data screening and extraction process

The lead review author (ED) will upload the search results and remove duplicates using EndNoteX8 (Clarivate Analytics). Search results will then be imported into Covidence (https://www.covidence.org), an online systematic review software, to conduct screening, data extraction and quality assessment. Two independent reviewers (ED and JB) will then screen titles and abstracts and classify studies (1) possibly meeting the eligibility criteria, (2) not meeting the eligibility criteria. Studies possibly meeting the inclusion criteria will then be obtained in full-text format. Two review authors will independently complete the full-text screening process (ED and JB). Any disagreement will be discussed and resolved with a third reviewer (AD). A PRISMA flow-chart diagram [34] will be used to report study selection process. Data extraction will be performed by two independent reviewers (ED and JB). For all studies, a grid will be used to extract data on the design, the geographic location, the context, the participants, and the objective of the included studies.

For all studies, a table inspired by the questioning formula what, why, who, when, and how that Colquhoun et al. [2] used to classify the characteristics of AF interventions with medical teams will serve the same purpose in our review. Since the object of interest constitutes a heterogeneous field of research [5], we chose to proceed with the steps of extraction, analysis, and presentation of results using reference frameworks previously selected by the research team to ensure a systematic process. Evidence from quantitative studies will be classified in a systematic way following the Donabedian’s quality of care triad [22]. Evidence from qualitative studies will be classified in a table inspired by Murray et al.’s framework [28]. This framework will be used to identify themes and classify perceived effects. Data on measured and perceived effects will be extracted from the results and discussion sections of the studies. Data will include statistical analyses, verbatim quotations, and narrative descriptive summaries of results. The extraction will draw on the method proposed by Sandelowski et al. [31] in order to preserve the methodological context of the findings.

### Quality assessment of included studies

The quality assessment of included studies will be carried out using the tool Mixed Methods Appraisal Tool (MMAT) [37]. This tool was developed specifically to assess the quality of items with a quantitative, qualitative, or mixed methods design [37]. The quantitative studies will be first categorized according to their characteristics and then assessed according to their respective characteristics, including the sampling strategy, the measuring instruments, and the response rate. For qualitative studies, the assessment criteria will include context, data sources, and data analysis. The evaluation criteria for the mixed methods studies will include the integration of the methods and the limitations presented. Two reviewers (ED and JB), for each of the criteria, will independently assign a score based on “yes,” “no,” “unspecified,” or “not applicable” responses. Any disagreement between the reviewers will be resolved by discussion or by requesting the assessment of a third reviewer (AD).

### Data analysis

Given the expected heterogeneity of quantitative data, the evidence on measured effects will be reported in a narrative form following a thematic analysis [38] based on Donabedian’s quality of care triad [22]. Qualitative data will be analyzed through the areas described in Murray et al. [28] framework. Data analysis of evidence from quantitative and qualitative studies will be conducted following the three-step narrative approach proposed by Popay et al. [33]. This approach is suited for inclusion of a wide range of research designs that generate both quantitative and qualitative findings on the effects of an intervention [33]. This approach encompasses (1) developing a preliminary synthesis, (2) exploring relationships in the data, and (3) assessing the robustness of the synthesis product [33]. A primary synthesis describing the patterns of direction and size of the effects will be undertaken from the extraction stage and considering the context and characteristics of the included studies [33]. This first synthesis will allow for an in-depth exploration of relationships between contexts, processes, and outcomes [33]. Patterns across studies will be identified by the reviewers in order to synthesis how characteristics and contexts influence the effects of A&F. Following this in-depth synthesis, its robustness will be assessed by considering the methodological quality of the included studies based on the MMAT [36].

### Data synthesis

Based on the segregated methodologies proposed by The Joanna Briggs Institute [17, 30] and based on Sandelowski et al. [29], an individual synthesis of quantitative and qualitative data will be performed. In this approach, a clear distinction between quantitative and qualitative evidence is required prior to the mixed method synthesis [17]. This first individual synthesis will be conducted following the approach proposed by Popay et al. [33] as previously described.

**Fig. 1:**
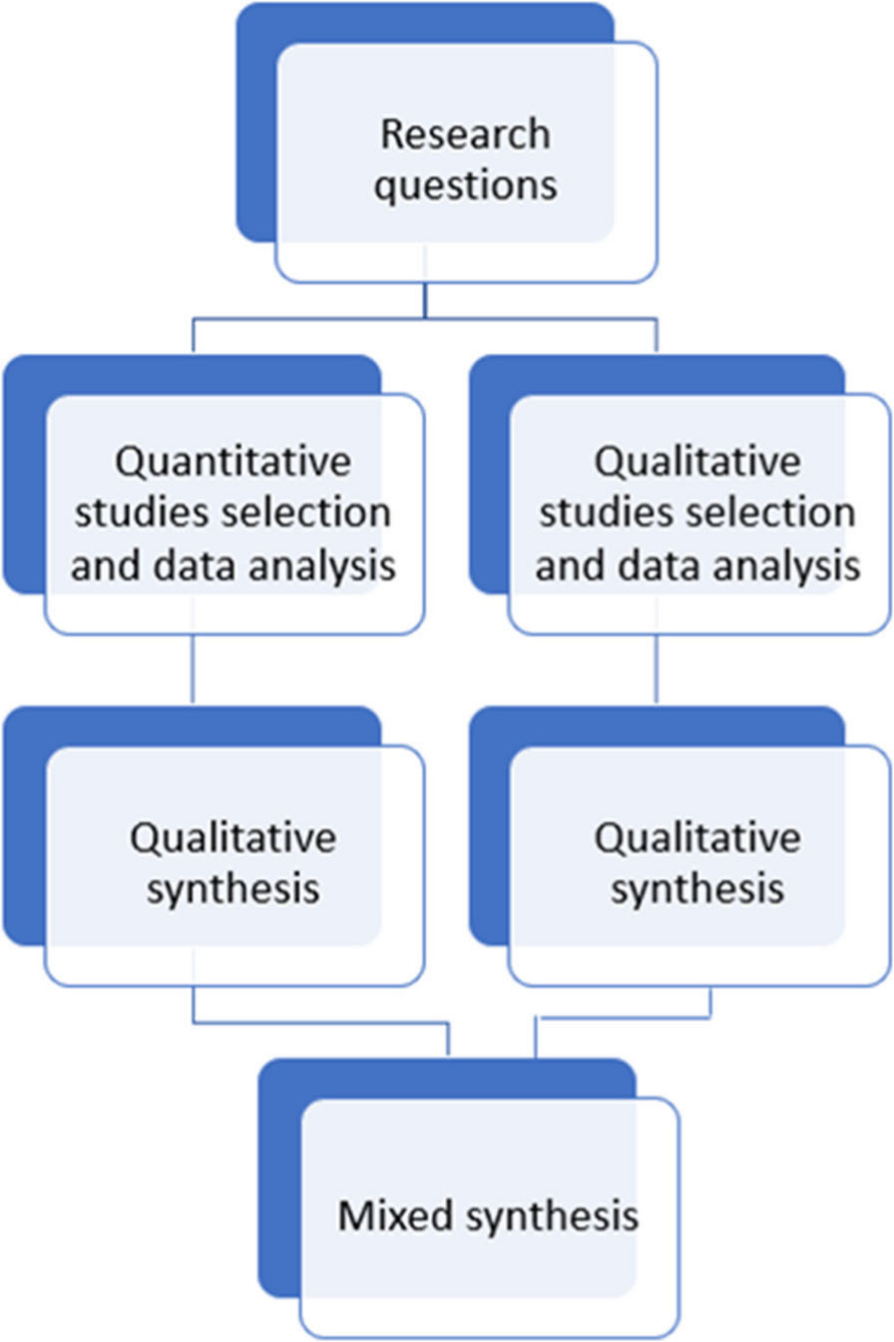
Data analysis plan. Inspired by the segregated analysis approach of The Joanna Briggs Institute [17]

A mixed method synthesis including all the generated findings will be then be conducted in order to study (1) the association between the A&F features and the measured or perceived effects and (2) the concordance between the measured effects and the perceived effects of A&F interventions with nurses. The combined findings will either support, contradict, or add to the quantitative and qualitative evidence [17]. This mixed method synthesis will follow a configuration approach [32, 39]. Using a configuration will allow to make connections between the evidence from both the quantitative and qualitative individual syntheses without merging them [17]. In this review, quantitative data are used to inform measured effects while qualitative data are used to inform perceived effects. Considering both types of effects have different implications for nursing performance, the findings will be used to complement rather than confirm each other [32]. Figure 1 presents the data analysis plan for this mixed method systematic review.

### Assessing confidence in the evidence

An assessment of the confidence in the evidence will be conducted from the recommendations made by The Joanna Briggs Institute [40–42]. These recommendations have been formulated in a complementary approach to that of the Grading of Recommendations Assessment, Development and Evaluation (GRADE) tool [42] and are based on the assessment of the coherence of the evidence according to the study specifications [42]. Given the heterogeneity of approaches to evaluating the effects of A&F interventions [5] as well as the inclusion of qualitative studies, this approach is favored in order to make appropriate recommendations. The levels of evidence proposed by The Joanna Briggs Institute [42], in a similar approach to that of GRADE, include the feasibility, relevance, effectiveness, and meaningfulness of the intervention under study. The latter relates more particularly to the evidence assessment for qualitative studies [42].

## Discussion

The primary objective of this mixed methods systematic review is to gather evidence on the measured and perceived effects of A&F interventions on nursing performance. Although systematic reviews on measured effects of A&F have been conducted on professionals’ practice and health outcomes [5], no review has focused on gathering effects specific to nursing practice. Furthermore, no review has aimed to assess evidence from qualitative as well as quantitative studies. We anticipate that combining these findings to qualitative studies will be highly relevant in providing insight on nurses’ perceptions on A&F and therefore to provide recommendations that can improve its design. This review will be valuable to health professionals interested in designing better suited A&F interventions for nurses. The strengths and the limits of the review, as well as recommendations for the implementation of A&F in nursing care will be discussed. One of the main limitations of this review is the heterogeneity in the literature regarding A&F in nursing. We anticipate A&F studies will take place in many different health settings, as well as in various forms and on different topics. We address this potential limitation by using frameworks specific to each type of data. The heterogeneity of the evidence will also be considered for the recommendations made following the evidence assessment.

## Additional file

Additional file 1: Search strategy for CINAHL. (DOCX 24 kb)

## Abbreviations

A&F: Audit and feedback
GRADE: Grading of Recommendations Assessment, Development and Evaluation
MMAT: Mixed methods appraisal tool
PRISMA: Preferred Reporting Items for Systematic Reviews and Meta-Analyses
