# Supplementary material for: Measured and perceived effects of audit and feedback on nursing performance: a mixed methods systematic review protocol
Source: Syst Rev. 2019 Feb 1;8:38. doi: 10.1186/s13643-019-0956-1 (PMC6357419; doi:10.1186/s13643-019-0956-1)
Supplement: Supplementary file 1 — Search strategy for CINAHL. (DOCX 24 kb) [file 13643_2019_956_MOESM1_ESM.docx]

|  | Audit and feedback | Nursing | Performance |
| --- | --- | --- | --- |
| CINAHL headings | (MH "Feedback") OR  (MH "Audit") OR  (MH "Nursing Audit") OR  (MH "Clinical Audit") | (MH "Nursing Role") OR (MH "Nurses+") OR (MH "Nursing Care+") | (MH "Quality of Health Care") OR  (MH "Quality Improvement ") OR  (MH "Clinical effectiveness") |
| Text words | feedback  "feed back"  "action planning"  audit  audits | Nurs* | Performance  Quality  Effectiveness  Security  Efficacy |

Search strategy for CINAHL

S1 (MH "Feedback") OR (MH "Audit") OR (MH "Nursing Audit") OR (MH "Clinical Audit") (26, 886)

S2 (MH "Nursing Role") OR (MH "Nurses+") OR (MH "Nursing Care+") (468,960)

S3 (MH "Quality of Health Care") OR (MH "Quality Improvement ") OR (MH "Clinical effectiveness") (100, 537)

S1 AND S2 AND S3 (717)

(4912)

S4 TI nurs* OR AB nurs* OR MW nurs* (812233)

S5 MW Feedback OR MW "Feed back" OR MW "Action planning" OR MW Audit OR MW Audits

S6 TI Feedback OR TI "Feed back" OR TI "Action planning" OR TI Audit OR TI Audits

S7 AB Feedback OR AB "Feed back" OR AB "Action planning" OR AB Audit OR AB Audits

S8 S5 OR S6 OR S7 (57545)

S9 TI performance OR TI quality OR TI effectiveness OR TI security OR TI efficacy

S10 AB performance OR AB quality OR AB effectiveness OR AB security OR AB efficacy

S11 MW performance OR MW quality OR MW effectiveness OR MW security OR MW efficacy

S12 S9 OR S10 OR S11 (794852)

S4 AND S8 AND S12
